# Ileal pouch-anal anastomosis for ulcerative colitis: 30-year analysis on surgical evolution and patient outcome

**DOI:** 10.1093/bjsopen/zrae111

**Published:** 2025-01-22

**Authors:** Gabriele Bislenghi, Antonio Luberto, Wout De Coster, Leen van Langenhoven, Albert Wolthuis, Marc Ferrante, Severine Vermeire, André D’Hoore

**Affiliations:** Department of Abdominal Surgery, University Hospitals Leuven, KU Leuven, Leuven, Belgium; Department of Abdominal Surgery, University Hospitals Leuven, KU Leuven, Leuven, Belgium; Department of Abdominal Surgery, University Hospitals Leuven, KU Leuven, Leuven, Belgium; Interuniversity Center for Biostatistics and Statistical Bioinformatics, KU Leuven, Leuven, Belgium; University of Hasselt, Hasselt, Belgium; Department of Abdominal Surgery, University Hospitals Leuven, KU Leuven, Leuven, Belgium; Department of Gastroenterology and Hepatology, University Hospitals Leuven, KU Leuven, Leuven, Belgium; Department of Chronic Diseases and Metabolism, KU Leuven, Leuven, Belgium; Department of Gastroenterology and Hepatology, University Hospitals Leuven, KU Leuven, Leuven, Belgium; Department of Chronic Diseases and Metabolism, KU Leuven, Leuven, Belgium; Department of Abdominal Surgery, University Hospitals Leuven, KU Leuven, Leuven, Belgium

## Abstract

**Background:**

Proctocolectomy with ileal pouch-anal anastomosis is the treatment of choice for patients with ulcerative colitis with medical refractory disease or dysplasia. The aim of this research was to describe the evolution of ileal pouch-anal anastomosis surgery and surgical outcomes over a three-decade interval in a high-volume referral centre.

**Methods:**

All consecutive patients undergoing ileal pouch-anal anastomosis for ulcerative colitis between 1990 and 2022 at the University Hospitals of Leuven were retrospectively included. Patients were divided into three interval arms (interval A 1990–2000, interval B 2001–2010 and interval C 2011–2022). The primary outcomes of interest were anastomotic leakage at 30 days and pouch failure.

**Results:**

Overall, 492 patients were included. The use of preoperative advanced therapies increased over time (*P* < 0.001). An increase in laparoscopic procedures (23.2% in interval A, 66.4% in interval B, 86.0% in interval C; *P* < 0.001) and a shift towards delayed ileal pouch-anal anastomosis (colectomy-first approach with delayed ileal pouch-anal anastomosis construction: 23.0% in interval A, 40.9% in interval B, 85.8% in interval C; *P* < 0.001) were observed. Anastomotic leakage rate decreased from 16.7% (interval A) to 8.4% (interval C) (*P* = 0.04). Delayed ileal pouch-anal anastomosis was the most relevant factor in limiting leakage (OR 0.49 (95% c.i. 0.27 to 0.87); *P* = 0.016). Median follow-up was 7.5 years (interquartile range 2.5–16). Cumulative pouch failure incidence was 8.2%, not significantly different between the three intervals (*P* = 0.580). Anastomotic leakage was the only significant risk factor for pouch failure (HR 2.82 (95% c.i. 1.29 to 6.20); *P* = 0.010).

**Conclusion:**

Significant changes in the management of ulcerative colitis patients occurred. Despite the widespread use of advanced therapies and the expanded surgical indications, anastomotic leakage rate decreased over time. In the context of a delayed ileal pouch-anal anastomosis, diverting ileostomy could be avoided in selected cases. Anastomotic leakage remains the most relevant risk factor for pouch failure. Pouch failure incidence remained stable over the years.

## Teaser

This study reviews the evolution of proctocolectomy with ileal pouch-anal anastomosis surgery and its outcomes over three decades at the University Hospitals of Leuven. Findings indicate an increase in laparoscopic procedures and delayed ileal pouch-anal anastomosis, leading to a significant decrease in anastomotic leakage rates over time, while anastomotic leakage was identified as a key risk factor for pouch failure.

## Introduction

Proctocolectomy with ileo pouch-anal anastomosis (IPAA) is the surgical treatment of choice for patients with ulcerative colitis with disease refractory to medications or complicated by dysplasia or cancer^1,2^. Although IPAA is associated with good patient satisfaction and high quality of life, the postoperative morbidity rate is 20–50% and long-term pouch failure (PF) occurs in 5–20% of patients^3^.

The landscape in the management of ulcerative colitis patients has undergone profound transformation over time, particularly in terms of timing of colectomy and operative strategy, due to significant advancements in both the surgical and medical field^4^. From a surgical perspective, these advancements have specifically focused on the implementation of minimally invasive approaches and cutting-edge technologies^5^. Consequently, there has been a minimization of the surgical trauma, with reduction of postoperative pain, surgical site infections, bowel obstructions and incisional hernias, faster recovery and improved cosmesis^6^. Significant changes have also occurred in the surgical technique to fashion the distal anastomosis^7^, the pouch configuration^8^, and the type of dissection and approach for the proctectomy^9,10^.

Concurrently, on the medical front, there has been a remarkable increase in the number of available medications for ulcerative colitis including biologicals and small molecules, which have been responsible for reducing colectomy rates over the past two decades. Nonetheless, approximately 30% of patients still require colectomy and debate is ongoing as to whether these medications are currently affecting short- and long-term outcomes of IPAA surgery^11,12^.

The objective of this study was to describe the evolution of IPAA surgery over a 30-year interval and to report rates and associated risk factors of anastomotic leakage (AL) and PF.

## Methods

### Design and patient selection

A monocentric retrospective study based on a pouch database was performed to identify all patients who underwent IPAA for ulcerative colitis between 1990 and 2022 at the University Hospitals of Leuven, Belgium (tertiary referral centre for inflammatory bowel disease). Data from 1990 to 2009 were collected retrospectively, whilst data from 2010 to 2022 were recorded prospectively in a dedicated institutional database). This study was approved by the Institutional Review Board of the University Hospitals Leuven (B322201213950/S53684) in the framework of our Crohn's disease and ulcerative colitis advanced researches registry. Informed consent was provided by all included patients.

Patient demographics included age, sex, body mass index (BMI), smoking status, indication for operation, American Society of Anesthesiologists score (ASA), diabetes mellitus, extraintestinal manifestation (EIM) including primary sclerosing cholangitis (PSC), time interval between ulcerative colitis diagnosis and first ulcerative colitis-related surgery, time interval between different surgical stages, and preoperative use and number of advanced therapies (biologicals and small molecules). Operative characteristics included number of surgical stages, surgical approach (open, laparoscopic, converted) and surgical technique for the proctectomy and IPAA construction ((transabdominal *versus* transanal (Ta-IPAA)), the plane of proctectomy dissection (total mesorectal excision (TME) *versus* close rectal dissection (CRD)), pouch configuration and type of distal anastomosis (hand-sewn, single-stapled, double-stapled). STROBE guidelines for observational studies were used^13^. Patients were divided into three interval arms: interval A from 1990 to 2000; interval B from 2001 to 2010; interval C from 2011 to 2022.

### Outcome and definitions

Anastomotic leakage (less than 30 days after surgery) was diagnosed radiologically by computed tomography or contrast-enema study showing contrast extravasation/sinus tract(s), or clinically at the time of reoperation on or digital examination of the ileoanal pouch under anaesthesia identifying an anastomotic defect. Pouch failure was defined as excision of the IPAA (followed by terminal ileostomy or redo IPAA) or permanent diversion (more than 2 years) with IPAA in place. Risk factors associated with the development of AL and PF were investigated. All procedures were performed by five different EBSQ (European Board of Surgical Qualification) qualified colorectal surgeons.

### Statistical analysis

Continuous data are presented as mean(s.d.) or median and interquartile range (i.q.r.); categorical data are presented as frequency and percentage. Comparisons between intervals were made using ANOVA, the Kruskall–Wallis test, the chi-square test and Fisher's exact test, as appropriate. Univariable logistic regression was used to identify possible risk factors for AL. Whenever the regression coefficients could not be estimated reliably due to (quasi-)complete separation of the data, Firth's penalized likelihood estimation for the logistic regression was used instead. A time-to-event analysis was performed to analyse PF. Because the occurrence of death precludes the occurrence of PF, the competing risk approach of Fine and Gray was used. To identify risk factors for PF, univariable Fine and Gray competing risk regressions were performed with death as a competing risk. Anastomotic leakage was modelled as a time-varying factor. Given the relative limited number of occurrences, it was deemed inappropriate to report results from a multivariable analysis. *P* values smaller than 0.05 were considered significant. The SAS system for Windows, version 9.4, was used for statistical analysis.

### Ethics

This research study was conducted retrospectively and prospectively (from 2010) from data obtained for clinical and research purposes. Approval was obtained from the ethics committee of University Hospitals Leuven. Informed consent was obtained from patients.

## Results

### Patients, disease characteristics and surgical details

**Fig. 1:**
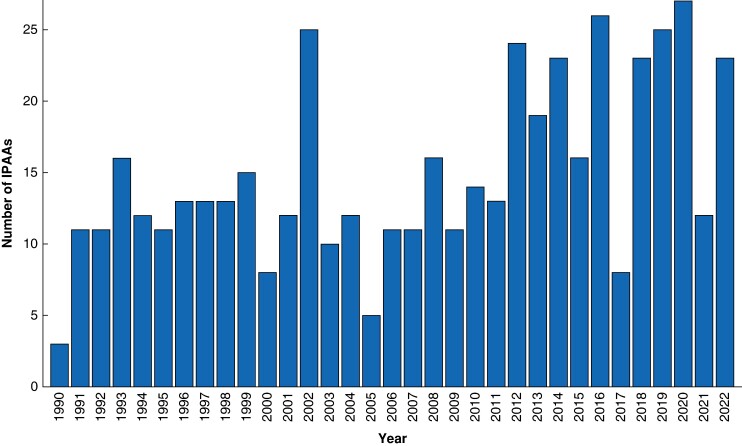
Annual volume of IPAAs at University Hospitals Leuven

Four hundred and ninety-two patients (209 female, 42.6%) were included. No patients from the consecutive cohort were excluded, however, for each feature, patients with missing data were not analysed. One hundred and twenty-six patients (25.6%) had IPAA surgery in interval A, 127 patients (25.8%) in interval B and 239 patients (48.6%) in interval C. The median number of IPAA operations/year was 13.0 (i.q.r. 11–19) (*Figure 1*).

IPAA, ileal pouch-anal anastomosis.

The mean age at first surgery was 39.8(13.6) years and significantly differed between the intervals (36.9(11.4) in interval A; 41.1(13.6) in interval B; 40.7(14.4) in interval C; *P* = 0.02). Mean BMI was 23.1(4.03) kg/m^2^ and also increased over time (22.7(4.6) in interval A; 22.4(3.94) in interval B; 23.5(3.95) in interval C; *P* = 0.06). Thirty-five patients (8.0%) were active smokers at the time of IPAA surgery. Concomitant PSC was present in 30 patients (6.2%).

The median interval between the diagnosis of ulcerative colitis and the first ulcerative colitis-related surgery was 72.0 months (i.q.r. 32.1–154.4) and increased between the intervals (53.4 (i.q.r. 29.0–111.8) in interval A; 75.9 (i.q.r. 28.8–169.0) in interval B; 76.7 (i.q.r. 35.0–167.0) in interval C), although the difference was not statistically significant (*P* = 0.06).

Seven patients (5.6%) underwent a one-stage IPAA in interval A, compared with 35.4% in interval B and 5.0% in interval C. A progressive decrease over the three time intervals in the rate of two-stage procedures was observed: from 71.4% in interval A to 23.6% in interval B to 9.2% in interval C. Altogether, 286 patients (58.1%) underwent a colectomy-first approach with delayed IPAA construction (modified two-stage or three-stage), with a significant difference over time (23.0% in interval A increasing to 40.9% in interval B and 85.8% in interval C; *P* < 0.001). Of these, 64 (13.0%) patients underwent a three-stage approach (19.1% in interval A *versus* 7.9% in interval B *versus* 12.6% in interval C). Overall, in 41.9% a diverting loop ileostomy after IPAA construction was used and this percentage changed significantly over time (dropping from 90.5% in interval A to 31.5% in interval B and 21.8% in interval C; *P* < 0.001).

Two hundred and fifty-seven patients (53.0%) received one or more advanced therapies before surgery. The number of advanced therapies used differed significantly over time, with six patients (4.8%) receiving equal to or greater than three advanced therapies in interval B increasing to 95 patients (40.8%) in interval C (*P* < 0.001). No patient received advanced therapies in interval A, 58 (46.0%) in interval B and 199 (85.4%) in interval C.

Reason for surgery was refractoriness to medical therapy in 339 of 486 patients (69.8%), acute severe colitis (ASUC) in 88 patients (18.1%), and ulcerative colitis-related cancer and dysplasia in 52 patients (10.7%). Reason for surgery did not change over time (*P* = 0.09).

**Fig. 2:**
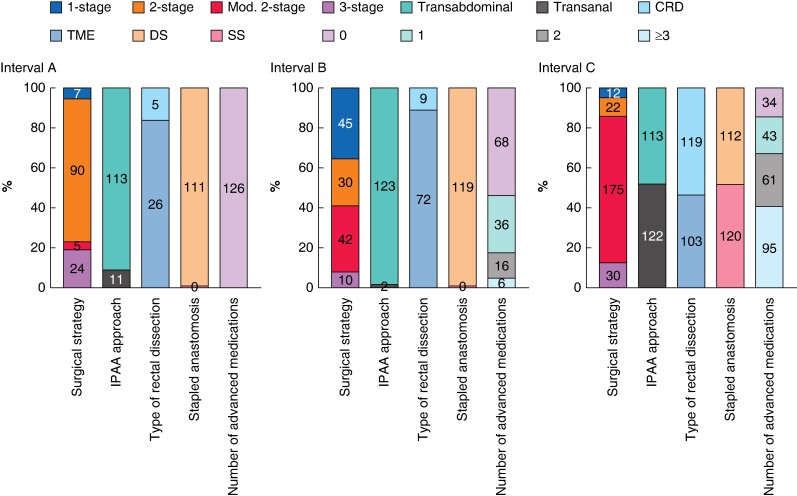
Variation over time of surgical strategies, IPAA approach, type of rectal dissection, anastomosis and preoperative use of advanced medications

IPAA, ileal pouch-anal anastomosis; SS, single stapled; CRD, close rectal dissection; TME, total mesorectal dissection; DS, double stapled.

IPAA surgery was performed laparoscopically in 312 of 482 cases (64.7%). The number of laparoscopic procedures increased over time (29 of 125 (23.2%) in interval A *versus* 81 of 122 (66.4%) in interval B *versus* 202 of 235 (86.0%) in interval C; *P* < 0.001). Conversion occurred in 22 (4.6%) patients. Overall, 122 of 484 (25.2%) underwent a Ta-IPAA. Since March 2015 (the date of the first Ta-IPAA at UZ Leuven), 117 of 154 patients (76.0%) received a Ta-IPAA. Overall, rectal dissection was performed via CRD in 133 of 334 patients (39.8%): a significant shift over the three intervals from a TME rectal dissection towards a CRD was observed (5 of 31 (16.1%) in interval A; 9 of 81 (11.1%) in interval B; 119 of 222 (53.6%) in interval C; *P* < 0.001). A stapled anastomosis was used in 463 of 488 patients (94.9%) of whom 120 (26.0%) were single-stapled. Patients’ characteristics and surgical details are extensively reported in *Tables 1* and *2* respectively. Surgical techniques are reported in *Fig. 2*.

**Table 1:** Patient and disease characteristics

Variable	1990–2000	2001–2010	2011–2022	Overall	
	(N = 126)	(N = 127)	(N = 239)	(N = 492)	*P*
Age at first surgery (years)*	36.9 (11.40)	41.1 (13.62)	40.7 (14.39)	39.8 (13.57)	0.0171
**Sex**					0.1771
Female	46/126 (36.51)	61/127 (48.03)	102/237 (43.04)	209/490 (42.65)	
Male	80/126 (63.49)	66/127 (51.97)	135/237 (56.96)	281/490 (57.35)	
BMI (kg/m^2^), mean (s.d.)	22.7 (4.6)	22.4 (3.94)	23.5 (3.95)	23.1 (4.03)	0.0559
**ASA**					<0.0001
I	6/36 (16.67)	25/119 (21.01)	2/239 (0.84)	33/394 (8.38)	
II	28/36 (77.78)	85/119 (71.43)	173/239 (72.38)	286/394 (72.59)	
III	2/36 (5.56)	9/119 (7.56)	64/239 (26.78)	75/394 (19.04)	
**Smoking status**					0.0013
No	52/78 (66.67)	88/125 (70.40)	172/237 (72.57)	312/440 (70.91)	
Current	15/78 (19.23)	8/125 (6.40)	12/237 (5.06)	35/440 (7.95)	
Past	11/78 (14.10)	29/125 (23.20)	53/237 (22.36)	93/440 (21.14)	
**No. of advanced therapies (before surgery)**					<0.0001
0	126/126 (100.0)	68/126 (53.97)	34/233 (14.59)	228/485 (47.01)	
1		36/126 (28.57)	43/233 (18.45)	79/485 (16.29)	
2		16/126 (12.70)	61/233 (26.18)	77/485 (15.88)	
≥3		6/126 (4.75)	95/233 (40.77)	101/485 (20.83)	
**PSC**					0.0147
No	125/126 (99.21)	116/126 (92.06)	217/236 (91.95)	458/488 (93.85)	
Yes	1/126 (0.79)	10/126 (7.94)	19/236 (8.05)	30/488 (6.15)	
**EIM (other than PSC)**					0.0472
No	110/126 (87.30)	101/126 (80.16)	211/236 (89.41)	422/488 (86.48)	
Yes	16/126 (12.70)	25/126 (19.84)	25/236 (10.59)	66/488 (13.52)	

Values are *n* (%) unless otherwise indicated. PSC, primary sclerosing cholangitis; ASA, American Society of Anesthesiologists; BMI, body mass index; EIM, extraintestinal manifestations.

**Table 2:** Surgical details

Variable	1990–2000	2001–2010	2011–2022	Overall	
	(N = 126)	(N = 127)	(N = 239)	(N = 492)	*P*
**Reason for surgery**					0.0916
CRC	2/124 (1.61)	5/126 (3.97)	12/236 (5.08)	19/486 (3.91)	
Refractoriness	95/124 (76.61)	86/126 (68.25)	158/236 (66.95)	339/486 (69.75)	
Dysplasia	3/124 (2.42)	9/126 (7.14)	21/236 (8.90)	33/486 (6.79)	
ASUC	24/124 (19.35)	25/126 (19.84)	39/236 (16.53)	88/486 (18.11)	
Other	–	1/126 (0.79)	6/236 (2.54)	7/486 (1.44)	
Interval diagnosis of UC and surgery (months), median (i.q.r.)	53.4 (29.0–111.8)	75.9 (28.8–169.0)	76.7 (35.0–167.0)	72.0 (32.1–154.4)	0.0559
Interval total colectomy and IPAA (months), median (i.q.r.)	0.0 (0.0–0.0)	0.0 (0.0–0.0)	3.5 (2.9–4.8)	2.7 (0.0–3.9)	<0.0001
Interval first surgery and IPAA (months), median (i.q.r.)	0.0 (0.0–0.0)	0.0 (0.0–0.0)	3.5 (2.9–5.0)	2.7 (0.0–4.0)	<0.0001
**Surgical strategy (N of stages)**					<0.0001
One	7/126 (5.56)	45/127 (35.43)	12/239 (5.02)	64/492 (13.01)	
Two	90/126 (71.43)	30/127 (23.62)	22/239 (9.21)	142/492 (28.86)	
Modified two	5/126 (3.97)	42/127 (33.07)	175/239 (73.22)	222/492 (45.12)	
Three	24/126 (19.05)	10/127 (7.87)	30/239 (12.55)	64/492 (13.01)	
**Colectomy-first + delayed IPAA**					<0.0001
No	97/126 (76.98)	75/127 (59.06)	34/239 (14.23)	206/492 (41.87)	
Yes	29/126 (23.02)	52/127 (40.94)	205/239 (85.77)	286/492 (58.13)	
**Intestinal diversion after IPAA construction**					<0.0001
No	12/126 (9.52)	87/127 (68.50)	187/239 (78.24)	286/492 (58.13)	
Yes	114/126 (90.48)	40/127 (31.50)	52/239 (21.76)	206/492 (41.87)	
**Surgical approach for IPAA**					<0.0001
Open	94/125 (75.20)	28/122 (22.95)	26/235 (11.06)	148/482 (30.71)	
Laparoscopic	29/125 (23.20)	81/122 (66.39)	202/235 (85.96)	312/482 (64.73)	
Converted	2/125 (1.60)	13/122 (10.66)	7/235 (2.98)	22/482 (4.56)	
**Surgical technique for IPAA**					<0.0001
Abdominal	126/126 (100)	127/127 (100)	119/236 (50.42)	372/489 (76.07)	
Transanal	–	–	117/236 (49.58)	117/489 (23.93)	
**Type of dissection**					<0.0001
Close rectal	5/31 (16.13)	9/81 (11.11)	119/222 (53.60)	133/334 (39.82)	
TME	26/31 (83.87)	72/81 (88.89)	103/222 (46.40)	201/334 (60.18)	
**Pouch configuration**					0.8056
J pouch	123/125 (98.40)	123/125 (98.40)	233/236 (98.73)	479/486 (98.56)	
S pouch	2/125 (1.60)	2/125 (1.60)	2/236 (0.85)	6/486 (1.23)	
H pouch	–	–	1/236 (0.42)	1/486 (0.21)	
**Type of anastomosis**					<0.0001
Hand-sewn	15/126 (11.90)	7/126 (5.56)	3/236 (1.27)	25/488 (5.12)	
Stapled	111/126 (88.10)	119/126 (94.44)	233/236 (98.73)	463/488 (94.88)	
**Type of stapled anastomosis**					<0.0001
DS	111/111 (100.0)	119/119 (100.0)	112/232 (48.28)	342/462 (74.03)	
SS			120/232 (51.72)	120/462 (25.97)	

Values are *n* (%) unless otherwise indicated. PSC, primary sclerosing cholangitis; ASA, American Society of Anesthesiologists, BMI, body mass index; CRC, colorectal cancer; UC, ulcerative colitis; ASUC, acute severe ulcerative colitis; TME, total mesorectal excision; DS, double stapled; SS, single stapled; IPAA, ileal pouch-anal anastomosis; i.q.r., interquartile range.

### Outcomes

#### Anastomotic leakage

**Fig. 3:**
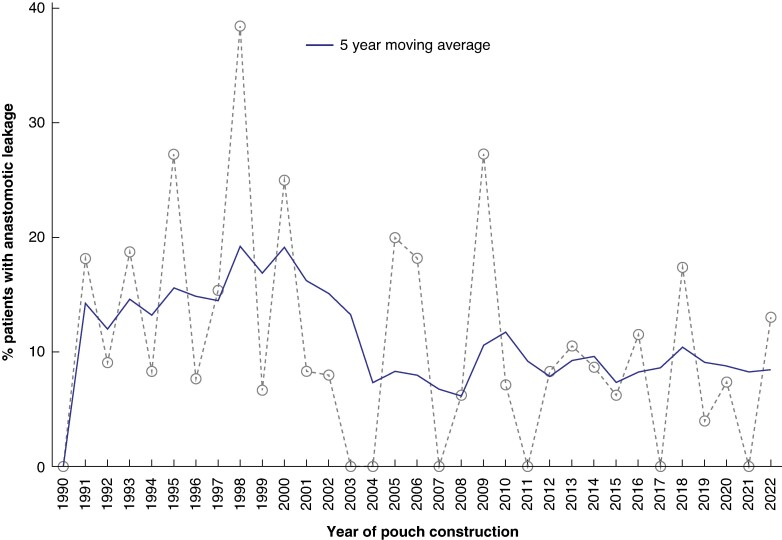
Percentage of patients with anastomotic leakage per year of pouch construction

Overall, AL occurred in 52 patients (10.6%) (21 patients (16.7%) in interval A; 11 patients (8.7%) in interval B; 20 patients (8.4%) in interval C) (*Figure 3*). Univariable logistic regression (*Table 3*) showed that the interval of IPAA construction was a risk factor for AL. IPAAs performed in interval C had a significantly lower risk of AL than those in interval A (OR 0.46 (95% c.i. 0.24 to 0.88); *P* = 0.02). Other variables associated with a lower risk of AL were the preoperative administration of advanced therapies (OR 0.52 (95% c.i. 0.29 to 0.93); *P* = 0.028), a staged surgical approach to IPAA (two-stage or modified two-stage or three-stage) (OR 0.31 (95% c.i. 0.16 to 0.60); *P* = 0.0006) and a colectomy-first approach with delayed IPAA construction (modified two-stage or three-stage) (OR 0.49 (95% c.i. 0.27 to 0.88); *P* = 0.016). On the other hand, age at first surgery was associated with an increased risk of AL (OR 1.025 for a 1-year increase in age (95% c.i. 1.004 to 1.047); *P* = 0.021).

**Table 3:** Risk factors for anastomotic leakage and pouch failure (univariable analysis)

Anastomotic leakage	Pouch failure					
Variable	OR*	95% c.i.	*P*	HR*	95% c.i.	*P*
Age at first surgery	1.025	1.004,1.047	0.0213	0.997	0.970,1.024	0.8226
Date of IPAA (year)	0.973	0.945,1.003	0.0795	1.026	0.989,1.064	0.1756
**Interval**						
B *versus* A	0.474	0.218,1.030	0.0594	1.293	0.604,2.766	0.5079
B *versus* C	1.038	0.481,2.241	0.9236	0.907	0.396,2.079	0.8183
C *versus* A	0.457	0.237,0.879	0.0190	1.425	0.623,3.259	0.4016
**Sex**						
Male *versus* female	1.460	0.800,2.666	0.2173	1.008	0.542,1.877	0.9798
BMI	1.015	0.925,1.115	0.7468	0.983	0.895,1.079	0.7132
**ASA**						
I *versus* II	0.166	0.009,2.959	0.2220	0.303	0.040,2.311	0.2492
I *versus* III	0.189	0.010,3.649	0.2699	0.488	0.050,4.805	0.5388
II *versus* III	1.134	0.457,2.812	0.7859	1.613	0.482,5.401	0.4383
**Smoking status**						
No *versus* current	0.750	0.162,3.465	0.7126	1.197	0.153,9.387	0.8640
No *versus* past	0.676	0.295,1.549	0.3545	0.541	0.233,1.254	0.1520
Past *versus* current	1.110	0.220,5.588	0.8997	2.214	0.264,18.558	0.4637
**PSC**						
Yes *versus* no	1.749	0.639,4.785	0.2763	1.445	0.456,4.575	0.5315
**Advanced therapies**						
Yes *versus* no	0.517	0.287,0.932	0.0283	1.473	0.759,2.862	0.2526
Number of advanced therapies	0.803	0.637,1.013	0.0640	1.145	0.916,1.431	0.2333
**Reason for surgery**						
Other *versus* ASUC	1.404	0.533,3.696	0.4921	1.372	0.370,5.079	0.6361
Other *versus* refractory	1.669	0.753,3.697	0.2068	0.941	0.332,2.669	0.9091
Refractory *versus* ASUC	0.841	0.397,1.781	0.6512	1.458	0.573,3.710	0.4293
Interval diagnosis-first surgery	1.106	0.849,1.439	0.4552	1.152	0.896,1.482	0.2703
**Staged IPAA**						
Yes *versus* no	0.309	0.158,0.604	0.0006	0.912	0.387,2.151	0.8337
**Surgical strategy**						
2-stage *versus* 1-stage	0.386	0.175,0.848	0.0178	0.627	0.239,1.647	0.3433
2-stage *versus* 3-stage	2.401	0.670,8.605	0.1787	0.356	0.153,0.828	0.0164
2-stage *versus* mod 2-stage	1.262	0.619,2.573	0.5221	0.645	0.293,1.418	0.2751
3-stage *versus* 1-stage	0.161	0.044,0.587	0.0057	1.760	0.631,4.910	0.2802
3-stage *versus* mod 2-stage	0.526	0.150,1.836	0.3135	1.810	0.748,4.379	0.1883
mod 2-stage *versus* 1-stage	0.306	0.145,0.644	0.0018	0.973	0.369,2.566	0.9552
**Delayed IPAA**						
Yes *versus* no	0.489	0.273,0.875	0.0160	1.676	0.899,3.126	0.1045
Interval total colectomy-IPAA	1.262	0.620,2.567	0.5207	1.187	0.680,2.074	0.5462
**Intestinal diversion after IPAA**						
1 *versus* 0	0.710	0.389,1.295	0.2640	0.912	0.477,1.745	0.7816
Interval-first surgery-IPAA	1.175	0.665,2.079	0.5786	1.171	0.813,1.686	0.3964
**Surgical approach pouch**						
Laparoscopic *versus* converted	2.233	0.290,17.186	0.4403	0.509	0.164,1.579	0.2421
Laparoscopic *versus* open	0.681	0.372,1.244	0.2116	0.790	0.410,1.520	0.4796
Open *versus* converted	3.280	0.418,25.742	0.2585	0.644	0.202,2.051	0.4567
**Surgical technique IPAA**						
Transanal *versus* transabdominal	0.838	0.416,1.688	0.6205	0.7506	0.258,2.183	0.5985
**Rectal dissection type**						
Close rectal *versus* TME	1.538	0.582,4.064	0.3855	1.398	0.455,4.298	0.5587
**Pouch type**						
S or H *versus* J	1.400	0.165,11.852	0.7577	3.981	0.688,23.043	0.1231
**Type of anastomosis**						
Hand-sewn *versus* manual	1.391	0.319,6.076	0.6605	2.784	0.370,20.964	0.3202
**Stapled anastomosis type**						
SS *versus* DS	0.889	0.448,1.764	0.7362	0.886	0.335,2.342	0.8074
**Anastomotic leak**						
Yes *versus* no				2.824	1.286,6.202	0.0097

Interval A 1990–2000; interval B 2001–2010; interval C 2011–2022. *Odds ratios have been calculated based on univariable logistic regression; hazard ratios based on univariable Fine and Gray regressions for competing risk for time-to-pouch failure. CRC, colorectal cancer; UC, ulcerative colitis; ASUC, acute severe ulcerative colitis; TME, total mesorectal excision; DS, double stapled; SS, single stapled; IPAA, ileal pouch-anal anastomosis; OR, odds ratio; HR, hazard ratio.

#### Pouch failure

**Fig. 4:**
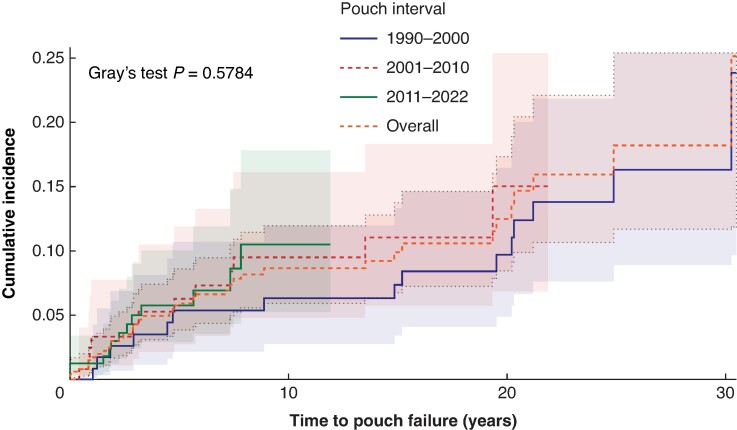
Cumulative incidence curve for overall and per interval incidence of pouch failure

Over a median follow-up of 7.5 years (i.q.r. 2.5–16), PF occurred in 40 patients (8.2%). Of them, 10 patients (25.0%) had a redo-IPAA and 30 patients (75.0%) a definitive ileostomy. In 22 patients (55.0%) a pouch excision was performed. The reason for PF was respectively pouchitis (25.0%), chronic pelvic sepsis (37.5%), poor IPAA function (15.0%), Crohn's disease (10.0%), or dysplasia/adenocarcinoma (5.0%). In 7.5% of patients the reason was unknown (missing data). The cumulative incidence curves of PF were not significantly different between the three interval arms (*P* = 0.58) (*Fig. 4*).

In univariable competing risk analyses, only AL came up as a significant risk factor for PF (HR 2.82 (95% c.i. 1.29 to 6.20)).

## Discussion

This comprehensive 30-year series of 492 consecutive patients who underwent IPAA for ulcerative colitis allowed assessment of incidence and risk factors associated with AL and PF, as well as their trends over time. It also offered the distinctive opportunity to observe the evolving approaches in managing ulcerative colitis patients, including the use of advanced therapies, surgical strategies and techniques. In the present series, the annual number of IPAA procedures showed a rising trend over time. During the last few years, a stable annual volume of equal to or greater than 20 procedures was maintained, in accordance with the requirements of European Crohn's and Colitis Organisation guidelines on centralization of IPAA surgery, which has been clearly linked to a reduction in postoperative complications^14,15^.

In the present series, AL and PF occurred in 10.6% and 8.2% of patients (median follow-up 7.5 years (i.q.r. 2.5–16)), in line with what was reported in the existing literature, where the AL and PF rate after IPAA respectively ranges between 5–20% and 3–14%^16^. A significant decrease of AL was observed over time: from 16.7% in interval A to 8.4% in interval C. This contrasts with the finding of a recent nationwide study from Denmark in which a significant four-fold increase in the risk of pelvic sepsis was observed, rising from 2.5% in 1996 to 9.6% in 2013^17^. In the present study, the occurrence of AL was a significant risk factor for long-term PF (HR 2.82; *P* = 0.01). Previous reports have highlighted the strict association between pelvic sepsis and PF. Nevertheless, only non-healing leaks evolving in chronic fistulas seem to be responsible for chronic pelvis sepsis and PF^16,18^. Therefore, early and effective treatment of AL remains crucial in preventing the formation of chronic pouch fistula limiting the burden of PF^19^.

The declining rate of AL in the present study was strictly associated with some other remarkable trends in the perioperative medical and surgical care of patients, primarily a progressive shift towards staged IPAA procedures^20^. The rate of colectomy-first approaches increased significantly from 23% in interval A to 85.8% in interval C. Delaying IPAA construction to a second surgical step (modified two- or three-stage procedure) seems to reduce the postoperative morbidity rate^21^. This was confirmed by the present data, highlighting a protective effect on AL rate when a colectomy-first approach was chosen (7.7% *versus* 14.6%; OR 0.49). This beneficial effect could be explained by the simultaneous withdrawal of all ulcerative colitis-related medications with the possible detrimental effect on wound healing and by the improvement of patients’ nutritional status^22^.

On the other hand, it remains unclear whether a diverting stoma should be used at the time of the IPAA construction, and in particular, whether a modified two-stage approach is preferable to a more prudent three-stage approach^23^. On this point, conflicting results have been reported. Nationwide data from Denmark indicated that non-diversion after IPAA leads to a 1.63 increased risk of IPAA failure and a 2.2 higher chance of impaired bowel function^14^. On the other hand, in a recent Canadian large retrospective series, routine diversion following delayed IPAA construction was not associated with a reduction in the incidence of IPAA-related sepsis (adjusted OR 0.79: 95% c.i. 0.53 to 1.17; *P* = 0.24) or PF (HR 0.64: 95% c.i. 0.39 to 1.07; *P* = 0.09)^24^. Moreover, in a multicentric study including more than 600 patients, de-functioning stoma was an independent risk factor for postoperative small bowel obstructions. In the present series, no difference for AL and PF was observed between a modified two-stage and a three-stage approach, possibly suggesting that diverting ileostomy may be safely avoided in selected patients^25^.

Older patients exhibited a higher likelihood of developing AL (OR 1.025; *P* = 0.02). Additionally, in the present series, a rise in the mean age of patients undergoing IPAA as well as BMI, simultaneous diagnosis of PSC and ASA score was depicted over the years, reflecting the probable tendency toward broadening the indications for IPAA surgery. Although this tendency could have potentially resulted in less stringent patient selection compared with earlier years, surgical outcomes were not affected^26^. The rise in the mean age of patients undergoing IPAA may be attributed to a longer disease duration, particularly when comparing patients operated on during the first decade to those in the later intervals of the cohort.

The present study effectively captured the progressive and exponential growth of available therapies for the treatment of ulcerative colitis (*P* < 0.001). About 40% of patients operated on in interval C received equal to or greater than three advanced medications compared with 5 and 0% in intervals B and A respectively. Interestingly, this remarkable trend was not paired with a parallel increase in disease duration before surgery, which remained stable (76 months) over the last 20 years. It could be that the preoperative exposure to an increasing number of different drug therapies including Anti-tumor necrosis factor, Vedolizumab, Ustekinumab and more recently, Tofacitinib, Ozanimod and Upadacitinib, would eventually have a detrimental effect on surgical outcomes. However, the contributing role of these advanced medications on the recently reported rising trend of pelvic sepsis, pouchitis and PF remains at the moment unproven and suppositional^17,27^. In the present series, a protective effect of advanced therapies on AL (OR 0.52, *P* = 0.02) was observed, although this effect disappeared after correction for time (*P* = 0.30). The indication for surgery did not shift over time from medically refractory disease to more cases of dysplasia or cancer, contrary to observations in other recently published studies. This discrepancy may be explained by differences in cohort characteristics, the type of operation performed (with only IPAA patients included in the present series), and the inclusion of incidental dysplasia/colorectal cancer identified in the pathological resection specimen but not known before surgery^28^.

With regard to specific technical aspects of IPAA surgery, apart from the already well documented shift towards laparoscopy, J pouch configuration and mechanical anastomosis, a major breakthrough in the present series emerged with the introduction of Ta-IPAA^9^. This ran in parallel with the implementation of CRD and the single-stapled anastomosis technique, contributing to a further decrease in surgical invasiveness^29^. Since 2015, about 75% of patients have undergone a laparoscopic (single incision laparoscopic surgery) + Ta-IPAA with CRD and single-stapled anastomosis. Risk analyses did not reveal any association between any of these surgical aspects and postoperative outcomes, leaving the debate on their impact on surgical outcomes still open.

This study has some limitations. It is a retrospective study with some retrospective data, although data from 2010 were recorded prospectively and reflected a better outcome for AL. Data on intestinal and sexual function and quality of life (not presented) were recorded only for patients operated on in more recent years, precluding any comparison between intervals. Some variables are missing data. Moreover, although this is one of the largest European single-centre series on IPAA for ulcerative colitis, the limited number of events for the outcomes considered did not allow multivariable analyses of potential risk factors for AL and PF. Finally, data on individual surgeon volume are lacking, which may represent a more reliable parameter of surgical expertise.

In conclusion, the present study depicted several important changes in IPAA surgery and in the perioperative management of ulcerative colitis patients over the last 30 years. Delayed pouch construction was the most relevant factor in limiting postoperative complications. Despite the increased rate of modified two-stage procedures, the exposure to a higher number of advanced therapies, and the expanded surgical indications, a decline over time in AL was clearly documented. Anastomotic leakage remained the most relevant risk factor for PF. The impact of some technical surgical aspects of IPAA on postoperative outcomes remains unclear and deserves further investigation.

## Data Availability

Institutional database. The data underlying this article will be shared on reasonable request to the corresponding author.
